# Parietal Alpha Oscillatory Peak Frequency Mediates the Effect of Practice on Visuospatial Working Memory Performance

**DOI:** 10.3390/vision6020030

**Published:** 2022-05-31

**Authors:** Riccardo Bertaccini, Giulia Ellena, Joaquin Macedo-Pascual, Fabrizio Carusi, Jelena Trajkovic, Claudia Poch, Vincenzo Romei

**Affiliations:** 1 Centro Studi e Ricerche in Neuroscienze Cognitive, Dipartimento di Psicologia, Alma Mater Studiorum—Università di Bologna, Campus di Cesena, 47521 Cesena, Italy; riccardo.bertaccini2@studio.unibo.it (R.B.); giulia.ellena@iit.it (G.E.); joamac12@gmail.com (J.M.-P.); fabrizio.carusi@studio.unibo.it (F.C.); jelena.trajkovic2@unibo.it (J.T.); 2 Center for Neuroscience and Cognitive Systems@UniTn, Istituto Italiano di Tecnologia, 38068 Rovereto, Italy; 3 Departamento de Psicología Experimental, Procesos Cognitivos y Logopedia, Universidad Complutense de Madrid, 28223 Madrid, Spain; 4 Departamento de Educación, Universidad de Nebrija, 28015 Madrid, Spain; cpoch@nebrija.es; 5 IRCCS Fondazione Santa Lucia, Via Ardeatina, 306/354, 00179 Roma, Italy

**Keywords:** working memory, oscillations, theta, alpha, individual peak frequency, inverse efficiency score, practice

## Abstract

Visuospatial working memory (WM) requires the activity of a spread network, including right parietal regions, to sustain storage capacity, attentional deployment, and active manipulation of information. Notably, while the electrophysiological correlates of such regions have been explored using many different indices, evidence for a functional involvement of the individual frequency peaks in the alpha (IAF) and theta bands (ITF) is still poor despite their relevance in many influential theories regarding WM. Interestingly, there is also a parallel lack of literature about the effect of short-term practice on WM performance. Here, we aim to clarify whether the simple repetition of a change-detection task might be beneficial to WM performance and to which degree these effects could be predicted by IAF and ITF. For this purpose, 25 healthy participants performed a change-detection task at baseline and in a retest session, while IAF and ITF were also measured. Results show that task repetition improves WM performance. In addition, right parietal IAF, but not ITF, accounts for performance gain such that faster IAF predicts higher performance gain. Our findings align with recent literature suggesting that the faster the posterior alpha, the finer the perceptual sampling rate, and the higher the WM performance gain.

## 1. Introduction

Working memory (WM) refers to the ability to temporarily store and manipulate limited amounts of information over a short period of time [1,2,3]. Following seminal findings from clinical research [4,5,6], a modularized multicomponent model was devised to account for all the different pieces of information working memory has to cope with [7,8], wherein non-verbal (e.g., visual and spatial) material pertains to specific and independent sub-systems. More recent neuroimaging studies concur in suggesting that such a compartmentalized visuospatial module might have some definite neural underpinnings, which correspond to the so-called frontoparietal network [9,10,11,12,13,14,15,16,17]. Indeed, the dorsolateral prefrontal cortex (dlPFC) and the posterior parietal cortex (PPC) have been proved to activate during a wide range of WM-related paradigms, sometimes showing slight lateralization toward the right (rather than left) hemisphere during visuospatial (as compared to verbal) tasks [18,19,20].

Whereas the contribution of dlPFC has been linked to top-down control mechanisms intrinsic to the central executive system, PPC is thought to underlie many processes associated with storage capacity, attentional deployment, and active manipulation of information [21,22,23,24]. These findings are in line with pieces of evidence gathered from patients suffering from parietal damage [25,26,27], who often display significant WM deficits, not to mention the severe impairment of spatial attention characterizing individuals affected by neglect syndrome, following right parietal lesions [28,29,30]. Besides all the clinical and neuroimaging data mentioned so far, electrophysiological inquiries have also highlighted the key role played by parietal areas in driving WM performance. For instance, frontoparietal oscillations within the theta band reported during WM tasks have been construed as a mechanism serving the prioritization and retention of relevant stimuli [20,31,32,33,34,35,36,37,38,39,40,41], while alpha activity over the same cortical regions should enhance ipsilateral performance by suppressing contralateral irrelevant information [33,38,41,42,43,44,45,46,47]. Similar conclusions have been reached by adopting non-invasive brain stimulation (NIBS) techniques as well [33,48,49,50,51,52,53].

Interestingly, the greater part of the above-mentioned EEG findings was collected via metrics such as spectral power, interregional phase-coupling, or electroencephalographic coherence, whereas other indices, namely band-specific individual peak frequencies, went often overlooked. Indeed, the literature regarding the role of theta and alpha peak frequencies is still poor, despite the relevance of such biomarkers in many influential theories concerning WM and attention [54,55]. Recently, a few studies have been carried out to shed some light on the matter. On the one hand, deceleration of parietal theta rhythms induced by transcranial alternating currents stimulation (tACS) has been shown to be beneficial to WM [49,51,52]. Conversely, externally induced acceleration of posterior alpha rhythms appeared to boost not only spatial WM performance [56] but also visual perception, probably by inducing a finer sampling rate [57,58,59,60,61,62]. Since frequency-specific peaks (especially in the alpha band) are known to be a rather stable neurophysiological trait that correlates with perceptual and cognitive abilities [49,61,63,64,65], it is legitimate to question whether individual alpha/theta peak frequencies (IAF/ITF) might help us to predict changes in WM performance as a function of practice.

Whereas extensive literature outlines that a general improvement in performance appears to correlate with large numbers of training sessions [66,67,68,69,70], little has been investigated regarding the impact of the simple repetition of a task on WM performance. Studies focusing on such short-term protocols, which were typically carried out within a single session or over two consecutive days at most, yielded scattered pieces of evidence on the matter [71,72,73,74]. Moreover, the aforementioned studies relied on neuroimaging techniques such as fMRI or PET, rather than EEG, whenever they were set to unveil any possible correlation between changes in WM performance and brain activity. In fact, only two major works have attempted to detail the EEG correlates of practice-induced changes relative to WM performance [75,76]. Specifically, both studies proved that accuracy and RTs benefitted from practicing the task. Yet, while in McEvoy et al. (1998), the electrophysiological investigations concerned EEG evoked responses, only Gevins and colleagues (1997) focused on oscillatory activity within the alpha and theta band. Indeed, in Gevins et al. (1997), participants performed, in a randomized manner, verbal and spatial WM tasks that could be either easy or difficult. An enhancement in accuracy and a decrease in reaction times were reported in both conditions as a function of practice, which were coupled with different oscillatory underpinnings. A power increase in the theta band over midfrontal areas, as well as in the alpha band over occipital sites, appeared to unfold after (as compared to before) practicing the task. Notably, such increases proved to be stronger, respectively, in the theta band for difficult tasks and in the alpha band for easy tasks. To sum up, this study confirmed that repetition-driven improvements could be associated with precise patterns of EEG activity. However, such patterns relied on analyses focused on spectral power measures instead of band-specific peak frequencies.

Here, we specifically aim to clarify whether and how short-term practice (i.e., the simple repetition of a visuospatial WM task) may benefit WM efficiency as measured using the inverse efficiency score (IES), an index combining reaction times (RTs) and accuracy, by adjusting each participant’s mean reaction time according to their own accuracy rate [77,78]. Moreover, we will assess whether and to which extent this surmised effect could be predicted by parietal individual peak frequency in the alpha and theta bands. We did so by asking 25 healthy participants to perform a visuospatial change-detection task [79] during a first (baseline) session and a second (retest) session that took place half an hour apart. We hypothesize that performance should increase (i.e., IES should decrease) at retest as compared to baseline (i.e., as a function of practice). Furthermore, we expect that such change might be predicted somehow by either ITF or IAF, as measured over the right parietal cortex, in line with clinical and experimental evidence suggesting that the right hemisphere (especially over posterior sites) is likely to host cognitive and perceptual systems responsible for processing and retaining visuospatial information, as well as driving the attentional focus toward both hemifields [9,18,20,28,29,30,80,81,82,83,84].

## 2. Materials and Methods

### 2.1. Participants

All the experimental sessions were carried out at the Center for Studies and Research in Cognitive Neuroscience in Cesena. A preliminary analysis performed on G*Power (parameters: effect size f = 0.30; α error probability = 0.05; power = 0.80; number of groups = 1; number of measurements = 4) returned an optimal sample size of 24 subjects. As a result, 25 healthy adult volunteers (15 females, mean age 23.32 ± 2.92 s.d. years old), naive as to the purpose of the study, were recruited (mostly during the first months of 2021) from the student population. Written informed consent was obtained from all the participants before taking part in the study, which was conducted in accordance with the Declaration of Helsinki and approved by the Bioethics Committee of the University of Bologna (Prot. 140758, 9 October 2018). All participants reported no history of psychiatric or neurological disorder, nor any other counter-indication such as taking psychiatric drugs. No monetary compensation was provided for those who volunteered in the study.

### 2.2. Procedure

**Figure 1:**
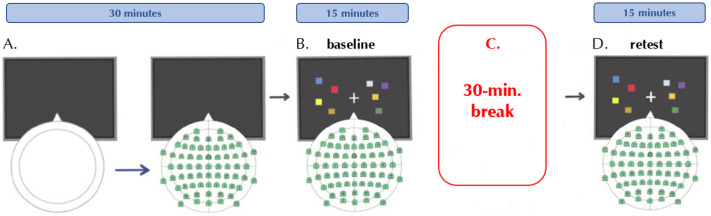
Schematic representation of the experimental session. (**A**) Setting up of the EEG cap. (**B**) Task performance at baseline. (**C**) 30 min break. (**D**) Task performance at retest.

The experimental session, which had a duration of approximately one and a half hours, progressed as follows. After signing the consent form, participants were seated on a comfortable chair in a sound-attenuated room. EEG cap was fitted, and electrophysiological activity was verified. Participants performed a change-detection paradigm task (baseline session) with a fixed load of 4 items to test for individual visual WM capacity. To identify the individual oscillatory peak, EEG analysis of retention periods (see task description in Section 2.3) was performed. The same change-detection task, along with EEG analysis of the retention periods, was performed around half an hour later (retest session). The structure of the experimental session can be appreciated in Figure 1.

### 2.3. Change-Detection Task

Visual stimuli of the task were displayed on a Relysis monitor (1280 × 1024, 85 Hz refresh rate) at a viewing distance of approximately 60 cm from the participant’s eyes. Stimuli were presented using Psychtoolbox v3 [85]; (http://www.psychtoolbox.org/. Accessed 10 December 2020), running under MATLAB R2016a (MathWorks) on a Windows machine.

**Figure 2:**
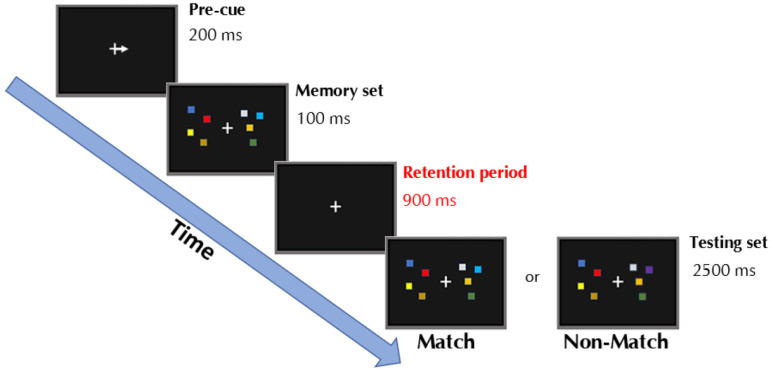
Schematic representation of the working memory (WM) change-discrimination task employed during each experimental trial.

In the task (Figure 2), an arrow was presented in the center of the screen for 0.2 s, indicating in which hemifield the to-be-remembered information was going to be presented. Subsequently, the memory set was presented for 0.1 s and consisted of four squares of different colors on each hemifield. The participants were instructed to only remember the squares cued by the arrow while ignoring the four squares presented on the opposite hemifield. A retention period of 0.9 s started, during which only a fixation cross was displayed, followed by a match/mismatch display for 2.5 s, during which the participant had to decide whether the new memory set presented matched (or not) the previously presented one by pressing two keys on a keyboard. On match trials, every square was of the same color as previously presented. On non-match trials, one of the squares of the relevant hemifield, cued by the arrow, changed color. After 2.5 s, the next trial started irrespective of whether the participant had responded. The task consisted of 160 trials, of which 40 were left-mismatched, 40 left matched, 40 right-mismatched, and 40 right-matched trials, presented in randomized order. Every 40 trials, a pause of few seconds was introduced to prevent participant fatigue.

### 2.4. EEG Recordings

EEG data were recorded using a 64 active Ag/AgCl electrodes cap arranged according to the 10-10 international system (ActiChamp, Brain Products, Gilching, Germany) and the software Brain Vision Recorder (Brain Products Italia Srl, Putignano, Italy) to record the electroencephalogram continuously throughout the whole experiment at 1000 Hz at each electrode. Electrodes were referenced online to the FCz electrode. Impedances of all electrodes were kept below 10 kΩ during the whole experiment. After the impedance check and before starting the recording, the EEG trace was visually inspected. This was to exclude any large artifacts from non-physiological sources, such as power lines, bad electrode contact, and broken electrodes. This check was repeated both for the first and the second recording session and while the EEG recording was ongoing.

### 2.5. Data Analyses

#### 2.5.1. Behavioral Data

As a measure of task performance, both accuracy rates (AR) and reaction times (RT) were computed for every participant in each session as a function of the cue pointing direction (left or right arrow cues). AR corresponded to the percentage of correct responses over the total amount of trials, namely, when the participant correctly reported if the testing set matched (or not) the memory set. On the other hand, RTs corresponded to the mean number of milliseconds (within the 2.5 s time window starting from the onset of the testing set) the participant took to press the response button in trials where a correct response was given. By implementing an automated MATLAB algorithm, we removed, for each participant, trials whose RTs exceeded more than two standard deviations from the participant’s mean RT: at baseline and retest, respectively, 4.4% (±1.2 s.d.) and 5.0% (±1.2 s.d.) of the total amount of trials were discarded. We then calculated an inverse efficiency score (IES), which is an index combining both RTs and accuracy by dividing each participant’s mean RT for their relative AR, according to the following formula [77,86,87]:

$$\mathrm{IES}=\frac{\mathrm{RT}}{\mathrm{AR}}$$

The advantage of using IES over ARs or RTs alone is that it better accounts for experimental conditions where higher accuracy rates are associated with faster reaction times (and vice versa) by down-weighting the contribution of slow RTs through an accuracy-based correction and might be therefore construed as an integrative measure of the overall efficiency displayed by a system [77,78].

#### 2.5.2. EEG Data: Preprocessing

EEG data were analyzed using Brain Vision Analyzer 2.0 (Brain Products Italia Srl). Electrodes were re-referenced offline to the average of all electrodes. Data were downsampled to 256 Hz. Continuous signals were segmented into epochs of 2100 ms, starting at 900 ms preceding the arrow-cue onset and for another 1200 ms after the cue onset. Data were then filtered with a high-band pass filter of 0.5 Hz and a low-band pass filter of 40 Hz (to minimize power line noise and motor artifacts) and, subsequently, baseline-corrected using a time window spanning from −600 to −300 ms before the arrow-cue onset. Moreover, residual artifacts contaminating the signal (eye blinks, eye movements, muscle contractions) were corrected by implementing an automated offline pipeline based on a linear regression method [88]. This procedure calculates the propagation factor between the eyes and each of the scalp electrodes and subtracts the corresponding proportion of the ocular activity from the waveform of each scalp site. This method allows subtracting the voltage due to muscular artifacts rather than rejecting trials with those artifacts. With these quality control steps (along with those described in paragraph 2.4), none of the participants had to be excluded.

#### 2.5.3. EEG Data: Peak Frequency Analyses

Preprocessed data were filtered to highlight our frequencies of interest (low cut-off, 3 Hz; high cut-off, 15 Hz). Subsequently, for every participant, power spectrum was extrapolated from each trial by implementing Fast Fourier Transform on the whole retention period (900 ms-long segments starting from the disappearance of the to-be-remembered array and ending at the onset of the match/mismatch display), with segments that were zero-padded to length of 1600 ms, resulting in a resolution of 0.1 Hz. Such participant-wise power spectra (sorted as a function of the testing sessions) were then averaged together to obtain power estimation relative, respectively, to the baseline and retest sessions. As a result, each participant’s IAFs and ITFs (both at baseline and retest) were extracted as the frequency peak within the alpha (7–13 Hz) and theta (3–7 Hz) band that showed the largest power estimate deviating from the 1/*f* scaling of EEG spectral activity [64,89]. Such detection strategy has been chosen over other approaches, namely, Center of Gravity (CoG) estimation [90], since it provides a more faithful and frequency-specific depiction of the physiological dynamics at work (as compared to the post hoc weighted reconstruction returned by CoG). Indeed, CoG has often been considered a valuable alternative to peak estimation whenever IAF had to be extrapolated from task-positive (rather than resting) EEG data due to event-related alpha suppression phenomena. However, more recent evidence shows that alpha activity might be just slightly attenuated or even enhanced during the completion of certain cognitive tasks [91,92]. Given that our analyses rely on data relative to retention periods, where alpha activity has been proven to be enhanced (and not decreased) [93,94], peak estimation (as opposed to CoG) appeared to be the most straightforward approach to implement.

Following multiple pieces of evidence regarding the role of frontoparietal sites in driving WM performance, we chose to analyze frequency peaks recorded from P4/P3 and F4/F3 electrodes, roughly corresponding to the right/left intraparietal sulcus and the posterior part of the right/left middle frontal gyrus [50,95,96,97,98]. As such, individual frequency peaks from the parietal (electrode P4, IAF_P4,_ and ITF_P4,_ experimental data) and frontal (electrode F4, IAF_F4_, and ITF_F4_, control data) lobes over the right hemisphere, as well as from parietal (electrode P3, IAF_P3_, and ITF_P3_, control data) and frontal (electrode F3, IAF_F3_, and ITF_F3_, control data) electrodes placed over the left hemisphere, were collected for the analyses.

### 2.6. Statistical Analyses

Statistical analyses were performed via jamovi 1.6 (the jamovi project, 2021). Concerning behavioral data, in order to assess whether inverse efficiency scores (IES) underwent changes as a function of practice, a repeated-measure ANOVA was performed, with side (left, right; according to the hemifield to-be-attended) and session (baseline, retest) as the within-subjects factors, and IES as the dependent variable. As for oscillatory peaks (IAFs/ITFs), in order to assess whether any practice-induced modulation occurred to them, two separate repeated-measure ANOVAs, for each frequency band (alpha and theta), were performed with hemisphere (left; right), electrode (frontal; parietal), and, most of all, session (baseline; retest) as the within-subjects factors.

Next, we assessed, via linear regression analysis, whether individual oscillatory peaks could account for interindividual variability in performance both at baseline and retest.

Importantly, given that behavioral data, but not oscillatory peaks, showed a significant effect of the session, we quantified the practice-induced gains in terms of performance by subtracting, for each participant, the mean IES scores at baseline from those at retest (ΔIES) and tested whether the practice-induced gains across individuals could be accounted for by their absolute individual frequency peaks both at baseline and retest by means of linear regression analysis.

## 3. Results

### 3.1. Inverse Efficiency Scores (IES)

**Figure 3:**
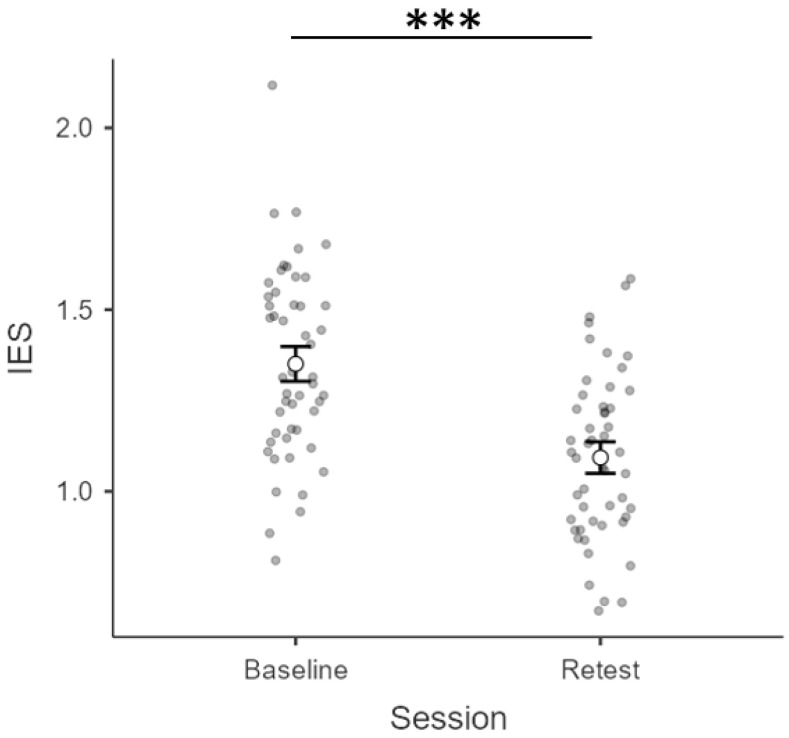
Estimated marginal means and error bars expressed as 95% standard errors. The graphs show side-collapsed observed scores relative to IES (*y*-axis) as a function of the testing session (*x*-axis): baseline (left) vs. retest (right). *** = *p* < 0.001.

We first checked for bias in terms of lateralized performance by comparing IES at baseline according to the location of the to-be-attended array (left vs. right hemifield). As expected, the paired-sample *t*-test returned no significant difference (*t_1,24_ = 0.609*; *p = 0.548*) between IES related to the left (*M = 1.36; S.E.M. = 0.06*) vs. right hemifield (*M = 1.34; S.E.M. = 0.04*). Subsequently, a repeated-measure ANOVA with session (baseline, retest) and side (left, right) as within-subject factors was computed. Results showed no main effect of side (*F_1,24_ = 0.185*; *p = 0.671*; *η^2^_p_ = 0.008*), nor any interaction of side × session (*F_1,24_ = 2.839*; *p = 0.105*; *η^2^_p_ = 0.106*), but a main effect of session (*F_1,24_ = 89.504*; *p < 0.001*; *η^2^_p_ = 0.789*). To better elucidate how the main effect of session was unfolding, we compared IES at baseline vs. retest (collapsing the factor side in each condition): IES at retest (*M = 1.09; S.E.M. = 0.04*) were lower than those at baseline (*M = 1.35; S.E.M. = 0.05*). Indeed, the paired-sample *t*-test run on side-collapsed IES at baseline vs. retest to further explore this change returned a significant difference (*p < 0.001, S.E.M. = 0.027; CI [0.20 0.31]*) along with a robust effect size (*Cohen’s d = 1.86*). To conclude, a general decrease in the inverse efficiency scores, indexing an improvement in WM performance, seemed to occur as a function of practice (independently of the to-be-attended hemifield). All the above-mentioned results are depicted in Figure 3**.**

### 3.2. Individual Frequency Peaks

We first checked for hemispheric differences both for anterior and posterior electrodes in averaged individual frequency peaks scores. Regarding the alpha band, paired-sample *t*-tests returned no significant interhemispheric difference for posterior electrodes ((*t_1,24_ = −1.453*; *p = 0.159*) between IAF_P3_ (*M = 10.3; S.E.M. = 0.261*) and IAF_P4_ (*M = 10.6; S.E.M. = 0.216*)) or anterior electrodes ((*t_1,24_ = 0.614*; *p = 0.545*) between IAF_F3_ (*M = 10.2; S.E.M. = 0.214*) and IAF_F4_ (*M = 10.2; S.E.M. = 0.226*)). Similarly, within the theta band, no significant interhemispheric difference was found for posterior and anterior electrodes (*all t_1,24_ values < −0.769*; *all p values > 0.05*) between ITF_P3_ (*M = 4.7; S.E.M. = 0.194*) and ITF_P4_ (*M = 4.8; S.E.M. = 0.170*), nor between ITF_F3_ (*M = 5.0; S.E.M. = 0.199*) and ITF_F4_ (*M = 5.1; S.E.M. = 0.241*).

**Figure 4:**
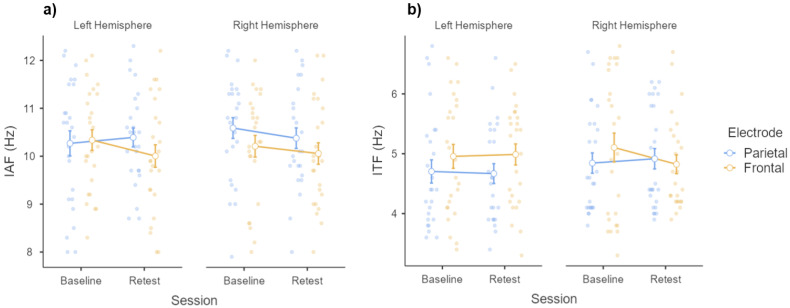
Estimated marginal means and error bars expressed as 95% standard errors. The graphs show observed scores relative to IAFs and ITFs (*y*-axes) as a function of testing session (*x*-axes, baseline vs. retest), sorted according to the hemisphere (left vs. right) and electrode location (parietal vs. frontal). Panel (**a**) shows IAFs (*y*-axis) as a function of the testing session (*x*-axis), recorded from parietal (blue dots) and frontal (orange dots) electrodes relative to the left (leftmost graph) and right (rightmost graph) hemisphere. Panel (**b**) shows ITFs (*y*-axis) as a function of the testing session (*x*-axis), recorded from parietal (blue dots) and frontal (orange dots) electrodes relative to the left (leftmost graph) and right (rightmost graph) hemisphere.

Next, in order to test whether individual peaks change as a function of session, two repeated-measure ANOVAs (one for each frequency band) were performed, with session (baseline; retest), hemisphere (left; right), and electrode (parietal; frontal) as within-subject factors. As for IAF, results showed no main effects of session, hemisphere, and electrode (*all F_1,24_ values < 2.396*; *all p-values > 0.05*; *all η^2^_p_ values < 0.091*), nor any significative interaction effect (*all F_1,24_ values < 1.632*; *all p-values > 0.05*; all *η^2^_p_ values < 0.064*). Likewise, ANOVA performed on ITFs returned no significant main effects of session, hemisphere, and electrode (*all F_1,24_ values < 2.845*; *all p-values > 0.05*; *all η^2^_p_ values < 0.106*), nor any significative interaction effect (*all F_1,24_ values < 1.165*; *all p-values > 0.05*; all *η^2^_p_ values < 0.046*). To sum up, oscillatory peaks within each frequency band displayed little variability, since they appeared not to change according to the electrode location, and, most of all, as a function of practice. All the above-mentioned results are depicted in Figure 4.

### 3.3. Brain Behavior Relationships

To assess whether parietal and frontal IAFs/ITFs relative to both hemispheres could predict any of the above-outlined behavioral patterns, we first tested whether individual oscillatory peaks could account for individual performance at baseline (and consistently at retest). Results showed no significant effect of IAFs/ITFs on IES scores both at baseline and retest (*all p-values > 0.05*).

Next, given that behavioral data but not oscillatory peaks showed a significant effect on the session, we tested whether IAFs/ITFs recorded at baseline, as well as at retest, from parietal and frontal electrodes relative to both hemispheres could predict performance gain (relative to the whole visual field) as measured with IES at retest relative to baseline (ΔIES). Results showed a significant effect of the right parietal IAFs recorded at baseline on ΔIES (*fitted regression model: ΔIES = 0.5404 − 0.0754* × *IAF_P4_ baseline*; *R^2^ = 0.359; F_1,23_ = 12.90; p = 0.002*). The same effect of right parietal IAFs on ΔIES was observed again at retest (fitted regression model: ΔIES = 0.3176 − 0.0554 * IAF_P4_ retest; *R^2^ = 0.190; F_1,23_ = 5.41; p = 0.029*). This effect was specific to hemisphere, site, and frequency. Indeed, frontal IAFs recorded from both the right and left hemispheres, as well as parietal IAFs recorded from the left hemisphere, did not exert a significant impact on ΔIES (*all p-values > 0.05*).

Similarly, no significant effect of frontal and parietal ITFs recorded from the left and right hemispheres on ΔIES could be observed (*all p-values > 0.05*).

**Figure 5:**
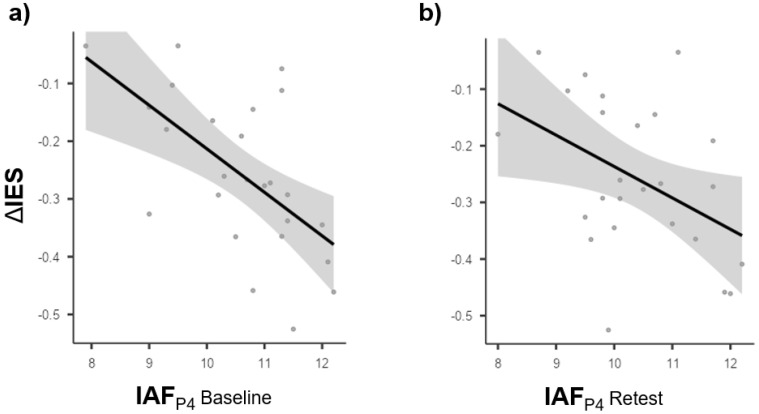
Scatterplots depicting the significant relationships unveiled by the linear regressions between individual alpha peak frequencies over the right parietal lobe and ΔIES. (**a**) Scatterplot of the relationship between changes in the inverse efficiency scores (ΔIES) at retest as compared to baseline (*y*-axis) and individual alpha peaks recorded over the right parietal lobe at baseline (IAF_P4_ baseline; *x*-axis); (**b**) Scatterplot of the relationship between changes in the inverse efficiency scores (ΔIES) at retest as compared to baseline (*y*-axis) and individual alpha peaks recorded over the right parietal lobe at retest (IAF_P4_ retest; *x*-axis).

To broadly summarize, it could be stated that neither IAFs nor ITFs (recorded at baseline and retest) can account for baseline performance; the performance gain as measured with ΔIES can be predicted by right parietal IAFs, independently of whether recorded at baseline or retest. Specifically, the faster the right parietal IAFs cycle, the more the IES decreases at retest (relative to baseline). All the above-mentioned results are depicted in Figure 5.

## 4. Discussion

In this study, we aimed to elucidate whether WM performance benefitted from the simple repetition of a change-detection task. Moreover, we were also interested in clarifying to which degree these putative improvements could be associated with the parietal individual oscillatory peak frequency in the alpha and theta bands. For this purpose, we recruited 25 healthy participants who performed a change-detection task twice: during a baseline session and (half an hour apart) a retest session, while individual peaks in alpha and theta frequency bands were extracted from the power spectra relative to the retention period during the baseline, as well as the retest session.

From a behavioral standpoint, the analyses met our predictions: IES, an integrative measure of task performance [77,78], significantly improved as a function of practice, akin to some previous findings relative to accuracy and RTs [71,73,75]. That is, even a simple repetition of the task was able to prompt noticeable changes in the performance relative to both the right and left hemifields. Let it be noted that these kinds of conclusions were mostly drawn by means of long-term studies [68,69,70], while evidence on the outcomes of short-term practice returned divergent suggestions [72,74]. However, this could be due to many factors, such as the limited number of studies focusing on the effect of short-term practice, their differences in terms of time devoted to practice, the overall design of the study, as well as the tasks, stimuli, and behavioral measures involved. We decided to keep these variables under control in two ways. On the one hand, adopting a combined index such as the IES (rather than accuracy and RTs alone) is thought to better track the resource consumption of a cognitive system and, consequently, its overall efficiency [78]. On the other hand, we chose to employ a change-detection task, which is a widely used and reliable paradigm to test visuospatial WM [70,79]. Moreover, given its relatively short length, it enabled us to collect a discrete amount of data through a simple test–retest design, thus preventing fatigue or loss of concentration as much as possible. These peculiar characteristics are also the reason why the change-detection task has been frequently adopted whenever it is necessary to lend some insight into the neural dynamics underlying WM. This consideration brings us to the second hypothesis behind our study, namely, that the observed behavioral improvements, ascribable to the repetition of the task, could be predicted by the speed of parietal oscillations in the alpha and theta band.

Indeed, clinical evidence [4,28,29,30] has paved the way for a functional model wherein the right parietal lobe acts as a crucial hub in driving the attentional focus toward both hemifields, leading many authors to agree with the “hemispatial” theory [82,83]. According to this conceptual framework, visuospatial attentional systems should be right-lateralized and yet capable of deploying the attentional focus toward the whole visual field and not just contralaterally [99,100]. In addition, right-lateralization was often found in WM tasks involving visuospatial (rather than verbal) stimuli [18,20]. Therefore, we ran some explorative analyses on individual alpha/theta frequency (focusing on oscillatory peaks recorded from right parietal sites) to elucidate whether they could be accounted for as some of the oscillatory signatures of such visuospatial vector, whose activities support spatial attention and visual WM. Interestingly, band-specific ANOVAs performed on oscillatory peaks to test for practice-induced changes yielded no significant results. Namely, both alpha and theta individual frequency peaks appeared not to be affected by the amount of practice, regardless of the hemisphere or the cortical site which they were recorded from. This piece of evidence is consistent with literature that likens frequency peaks to an individual trait with a high degree of stability [63,64]. Moreover, our data further extend existing knowledge on the matter, suggesting that such stability appears to be immune from short-term, practice-related effects as well.

In light of the aforementioned findings, brain–behavior relationships were investigated by comparing performance gain with individual frequency peaks, respectively, at baseline and retest. Results returned by the regression analyses depicted a clear pattern, where participants showing faster alpha rhythms (both at baseline and retest) over the right parietal cortex displayed a greater decrease in IES over the course of the experiment. In other words, alpha peak frequency over the right parietal cortex appeared to predict the extent to which participants benefitted from the repetition of the task in terms of performance. This is not surprising, considering that oscillatory activity in the alpha band has been linked to perceptual processing [57,101,102,103,104] and the efficiency by which distracting stimuli are dealt with [33,55,105]. This is because the efficacy of both functional phenomena is likely to depend on the number of alpha cycles fulfilled per second. This measure, in turn, determines the degree of resolution of each individual’s perceptual sampling rate [62,106] and might very well be regarded as a variable affecting WM performance [42,107,108,109,110], namely, the higher the frequency of the individual alpha peak is, the more the IES decreases. Since performance estimates gathered via IES account for the degree of efficiency by which a system draws upon its internal resources, the individual alpha peak might then be construed as the neurophysiological parameter ensuring enough cognitive flexibility to capitalize on the amount of practice. Indeed, the rate at which individual alpha peaks cycle, rather than driving the performance per se (as control analyses outlined), appears to determine how efficiently participants tap into the cognitive capabilities enabling them to profit from the repetition of the task. In line with recent evidence, such capabilities might regard the speed of information processing [64] and/or the extent to which individuals learn (as the task progresses) how distracting stimuli should be effectively dealt with [42,55,111]. Remarkably, both the alpha peaks at baseline and retest proved to predict participant ability to flexibly adapt to task demands (i.e., as a function of practice), further corroborating previously mentioned results regarding the trait-like features (e.g., stability) of such an electrophysiological index. Moreover, only individual alpha peaks over the right (and not the left) parietal lobe seem to be causally involved in determining the efficiency gain (relative to the whole visual field) over the course of the experiment, resembling many functional and topographical characteristics ascribed to the attentional vector claimed by advocates of the hemispatial theory [82,83]. Overall, these pieces of evidence depict a psychophysiological scenario where the speed of parietal alpha oscillations appears to represent a reliable biomarker of short-term practice outcomes. Whereas it remains to be further clarified whether this relationship applies to cognitive domains other than those regarding WM (i.e., the “far” transfer problem), the translational meaning of such effects needs to be accounted for. Specifically, preliminary screenings to assess parietal alpha frequency might help clinicians in predicting the effectiveness of short-term training protocols to be administered to patients suffering from attentional or cognitive impairments. As a result, more customized treatment strategies tailored to each patient’s electrophysiological traits would be enabled.

On the other hand, data relative to individual theta peak frequency appears to be inconclusive. Oscillatory activity in the theta band has been thought to sustain online maintenance of the to-be-remembered items during WM tasks [38,49,52,112,113,114]. According to the theta–gamma code theory [54,115,116], one would expect that individual theta peaks displaying slower frequency rates should leave more room for gamma spikes to nest in, thus enhancing WM capacity by virtue of a better phase-amplitude coupling. This seems to be at odds with our results since our analyses unveiled no such relationship. However, the patterns returned by our analyses may be alternatively interpreted. For instance, the fixed load of four items, corresponding to the average WM storage limit [3,117], may not represent a sufficient cognitive load to engage theta speed dynamics underlying storage capacity. Indeed, oscillatory activity in the theta band has been proved to increase during WM tasks in proportion to the difficulty or the cognitive load relative to the employed paradigms [33,75,118,119]. Moreover, a crucial role in top-down control and cognitive monitoring has also been ascribed to rhythmic activity between 3 and 7 Hz [120,121]. Taken together, these pieces of evidence suggest that our paradigm, both in terms of cognitive load and overall structure, was not sufficiently challenging to solicit a massive involvement of theta activity (as measured through frequency peaks over frontal and parietal sites) in support of performance. This is likely to be ascribed to the number of to-be-remembered items (corresponding to the average storage capacity, which was not increased during the course of the experiment), as well as the occurrence of the retest session, during which the completion of the task may have been easier (due to practice) than that relative to the baseline session, thus reducing the need for monitoring processes.

To summarize, the simple repetition of a change-detection task has proved to be beneficial to WM performance as assessed via IES. This improvement appears to be predicted by the individual alpha (but not theta) frequency recorded over the right parietal lobe, both at baseline and retest. Such patterns point to a functional role played by right parietal alpha peaks in facilitating the implementation of new and more efficient cognitive strategies developed over the course of the experiment as a function of practice.

## 5. Limitations

Despite the promising pieces of evidence provided in our study, some further issues need to be considered. Firstly, our sample size was relatively small, and a wider number of participants is needed in order to draw more solid conclusions about the topics we have investigated. On this same line of reasoning, participants in our experiment were mostly psychology students whose average age and years of scholarly education differ from those relative to the general population. As a result, not only a bigger but also a more heterogeneous sample size will be required for study replication. Besides matters concerning the size and demographics of the sample, a further potential limitation might regard the extent to which the above-described effects apply to other domains. That is, we cannot entirely rule out the possibility that our results could be task-specific and may not be witnessed when probed with different WM tasks or experimental paradigms designed to test other cognitive functions (the “near” and “far” transfer issue). Forthcoming study replications should take into account such considerations as well.

## Data Availability

The data presented in this study are available on request from the corresponding author.
